# New Aluminum Syntactic Foam: Synthesis and Mechanical Characterization

**DOI:** 10.3390/ma15155320

**Published:** 2022-08-02

**Authors:** A. M. Sánchez de la Muela, L. E. García Cambronero, L. F. Malheiros, J. M. Ruiz-Román

**Affiliations:** 1 Department of Geologic and Mining Engineering, Escuela Técnica Superior de Ingenieros de Minas y Energía, Universidad Politécnica de Madrid, 28003 Madrid, Spain; luis.gcambronero@upm.es (L.E.G.C.); josemanuel.ruizr@upm.es (J.M.R.-R.); 2 Associated Laboratory for Energy, Transport and Aeronautics, LAETA (PROA), Faculty of Engineering, University of Porto, 4200-465 Porto, Portugal; lfmf@fe.up.pt

**Keywords:** MMSF, CMF, aluminum, porous, marble, composite metal foam

## Abstract

Metal matrix syntactic foams (MMSF) are advanced cellular materials constituted by a system of a minimum of two phases, in which a dispersion of hollow particles is embedded by a continuous metal matrix. The incorporation of porous fillers favors the development of low-density materials with exceptional behavior for damping vibrations, impacts, and blast effects, shielding acoustic, thermal, and electromagnetic energies. There are three main techniques to produce them: infiltration casting technique (ICT), stir casting technique (SCT), and powder metallurgy technique (P/M). The first two techniques are used for embedding filler into lower melting point metallic matrices than fillers, in contrast to P/M. The present study demonstrates the feasibility of producing MMSF with components of similar melting points by ICT. The fillers were synthesized in-situ with aluminum and a natural foaming agent from wastes of Spanish white marble quarries. These novel aluminum syntactic foams (ASF) were mechanically characterized following the ISO-13314 and exhibited a porosity, plateau stress, and energy absorption capacity of 41%, 37.65 MPa, 8.62 MJ/m^3^ (at 35% of densification), respectively. These properties are slightly superior to equal porosity LECA ASF, making these novel ASF suitable for the same applications as LECA-ASF.

## 1. Introduction

The term composite metal foam (CMF) was introduced by Rabiei to refer to metallic foams with closed-cell porosity formed by two solid phases: a metallic phase of hollow particles and a continuous metallic phase that surrounds them known as fillers and matrix, respectively. This approach develops higher density and less porosity than conventional closed-cell metal foams (MF). However, CMF shows the advantage of being manufactured through almost the same techniques as metal matrix composites (MMC), exhibits similar behavior to MF, and can be fitted through an adequate selection of fillers and matrix properties [1,2]. The low density of CMF and the incorporation of closed-cell fillers make them adequate materials for applications where density is a limiting parameter or where thermal isolation, fire-proof sandwich core, electromagnetic shielding, acoustic and vibrations damping, and impact, blast, and ballistic protection are required [3,4].

The behavior of CMF and the suitability of the components’ properties promoted the development of a new kind of foam known as metal matrix syntactic foams (MMSF). The chemical composition of fillers is non-metallic, and their internal morphology is not limited to closed-cell monopore porosity. Efforts to reduce the costs of synthesizing MMSF have favored the incorporation of porous lightweight aggregates (LWA) such as expanded clay (EC), expanded perlite (EP) pumice, and vermiculite [5]. Metallic alloys commonly used in CMF and MMSF are based on Mg, Al, Pb, Ti, and Fe. These materials can be manufactured through three main routes: melt infiltration technique (MIT), stir casting technique (SCT), and powder metallurgy (P/M). The MIT consists of filling the open porosity between a bed of fillers with molten matrix promoting the infiltration through the mechanical, atmosphere, or inertial forces [6]. The SCT consists of creating a vortex in the melt and then pouring preheated filler into the vortex to favor homogeneous dispersion [7]. The P/M consists of mixing fillers and powders of the metallic matrix and then subjecting the mixture to a sintering process at a lower temperature than the matrix melting point. Casting techniques are employed for processing alloys with a melting temperature less than 900 C (Al, Mg, Zn, or Pb), while for higher melting temperatures (Ti and Fe), it is recommended to apply P/M [8].

Synthesizing aluminum syntactic foams (ASF) by infiltration routes and embedding porous fillers have been extensively studied by diverse research groups such as those of Friedler, Orbulov, Puga, and Bonabi. The first group studied EP-AlSi7Mg (AlSi7Mg: 7.2 wt% Si and 0.1 wt% Mg alloying and EP: silica ~75 wt%—alumina ~14 wt%) syntactic foams in terms of mechanical compression properties with and without heat treatments, and the effect of topology and diameter of fillers on the performance of SF has been also analyzed [9,10,11,12,13,14]. Alternatively, AlSi7Mg-embedding pumice particles [12] were also studied mechanically under a compressive quasi-static regime. Pumice is a volcanic rock with a chemical composition of ~75 wt% SiO_2_, ~14 wt% Al_2_O_3_, and other oxides: Na_2_O, K_2_O, CaO, Fe_2_O_3_. This particle exhibits an irregular topology that favors anisotropy in properties. This new approach exhibited a plateau stress and absorption energy capacity of 68.3 MPa and 24.8 MJ/m^3^, respectively. Subsequently, Wright and Kennedy were able to reduce the density of ASF to 1 g/cm^3^ by incorporating expanded glass fillers (EG, ~71 wt% SiO_2_, ~12 wt% Na_2_O, ~9 wt% CaO, ~2 wt% Al_2_O_3_, ~2 wt% MgO and other oxides) into AlSi12 alloy [15]. Compressive properties of AlSi7Mg-EG ASF were analyzed, and it was found that shrinkage phenomena on fillers improved the stress plateau from 15 to 26 MPa [14]. The mechanical behavior of EP and EG particles embedded by Zn alloy (ZA27) at temperatures ranging from 25 to 350 °C showed that EP has superior behavior to EG [16].

A great performance was reached by embedding a structured layer of perlite (EP) and activated carbon (AC) particles. Conventional (two layers) and multilayer bimodal ASF embedding consecutive layers of EP and AC were studied. Superior performance and higher densification strains were found compared to conventional single-layer EP-SF and AC-SF [17,18,19].

Diverse research groups, such as those of Bonabi, Puga, and Orbulov [20,21,22,23], have analyzed ASF embedding expanded clay particles (EC, silicon y alumina at 50 wt% and 15 wt%, respectively). The behavior of ASF constituted by AlSi5Cu1Mg and EC of 6 to 8 mm in diameter was studied, and a compressive stress and absorption energy capacity of 35.9 MPa and 18 MJ/m^3^ was reached [20]. By employing AlSi7Mg matrix alloy and EC particles ranging from 2 to 7.5 mm in size, the plateau stress and energy absorption capacity were 32.2 MPa and 32.2 MJ/m^3^, respectively [21]. EC fillers ranging from 4 to 11 mm in size have also been embedded by Al99.5 (99.5 wt% Al and 0.1% wt% Si) and AlSi9MgMn (88.8 wt% Al, 9.8 wt% Si, 0.3 wt% Mg, 0.8 wt% Mn, 0.1 wt% Fe, 0.2 wt% others). Mechanical properties in the Q-S regime ranged from 22.3 to 83.1 MPa in compressive stress, 17.0–95.6 MPa for stress plateau, 40–50% of densified, and 8.9–48.1 MJ/m^3^ of energy absorption capacity. Samples tested as-cast and embedding high-size fillers exhibited lower values [22]. Orbulov et al. showed that using EC nodules could reduce economic costs and potentially synthesize ASFs for the transport industry [23]. Subsequently, Kemeny et al. and Leveles et al. [24,25,26] demonstrated that using EC-ASF (AlSi12) as filler of an AlMgSi0.5 tube (ASF-FFT) improves the compressive performance. Furthermore, it was found that ex-situ filled tubes and bimodal ASF-FFT embedding EC in different sizes (2.5–3 and 3.5–4 mm) with the same volume ratio enhance the mechanical properties. Previously, Movahedi and Linul [27] analyzed the benefits of filling aluminum tubes with EC nodules fixed together with a polymer bonding agent. The capacity of energy absorption of the filled tubes increased by 24% in comparison with empty tubes.

Additionally, the group of Rabiei developed CMF through P/M and MIT routes using hollow steel spheres ranging from 1.4 to 3.7 mm in size. CMF with matrices alloys of AlSi7 and low carbon steel SS316L were tested at the quasi-static compressive regime and exhibited 67, 76, and 136 MPa of plateau stress and 32, 37, and 68 MJ/m^3^ of energy absorption capacity, respectively [28,29,30]. Posterior dynamic studies demonstrated excellent behavior of the mentioned CMFs as the core of sandwich panels for ballistic and blast protection [31,32]. It is interesting to note that the mechanical properties of AlSi7 and low carbon steel CMFs testing in the Q-S regime are in the range of the heat-treated and low-cost EC-ASF studied by Orbulov’s team.

The research groups of Vesenjak, Krstulovic-Opara, and Duarte have extensively studied aluminum expanded nodules foamed with TiH2 (APM). Compressive testing on APM made of AlSi7 and AlSi10 matrices ranging from 5 to 10 mm in size demonstrated great potential for their use in general engineering applications [33,34,35]. Diverse hybrid foam approaches were tested based on the APM potential, and its incorporation as a filler of polymer SF, aluminum tubes and to obtain open cell foam structures was found to be beneficial [36,37,38].

From the best knowledge of the authors, there are no studies about CMF and MMSF that incorporate spheroidal metallic fillers with a closed-cell porous internal morphology. Thus, the present work focuses on the production and the mechanical characterization of novel aluminum syntactic foam (ASF) obtained by casting routes. Mechanical testing has been analyzed according to the ISO-13314 standard, and low-cost EC-ASF have been used as reference material. The fillers of this novel ASF are nodules of expanded aluminum (EAn), which were foamed with milled rock wastes from white marble quarries. In addition, this approach provides the advantage of using mining industry wastes as raw materials and highly recyclable metallic components (aluminum), which favors limiting the excessive exploitation of natural sources.

## 2. Materials and Methods

### 2.1. Materials

The aluminum alloy grade 6063 (Al-Si-Mg) was used as a metal matrix, and it was provided as an extrusion product by Hydro Aluminum Iberia, S.A.U., Guadalajara, Spain. The chemical composition in wt% is 0.2–0.6 Si, 0.35 Fe, 0.10 Cu, 0.10 Mn, 0.45–0.9 Mg, 0.10 Cr, 0.10 Zn, 0.10 Ti, 0.15 other and balanced Al. It has a density of 2700 kg/m^3^, an elastic modulus of 68.8 GPa, and a melting temperature of 650 °C [39].

**Figure 1:**
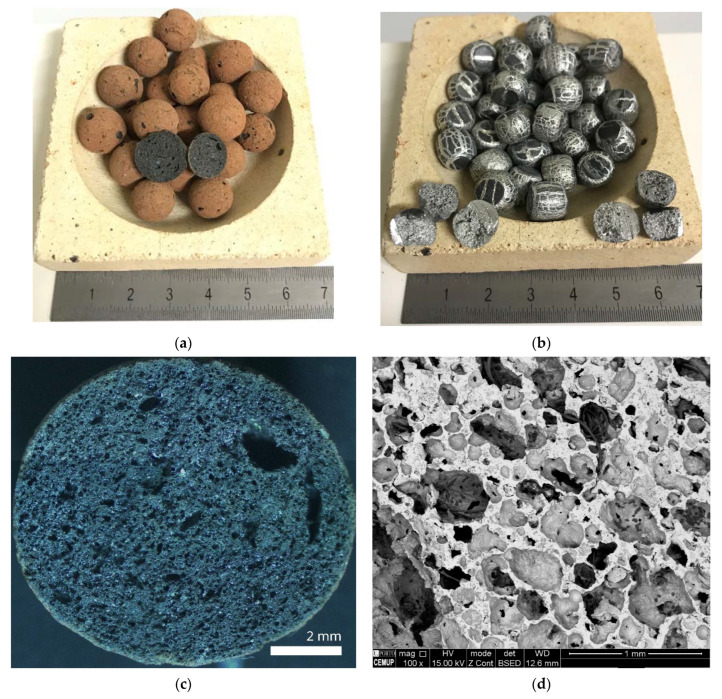
The physical aspect of particles: (**a**) ECn and (**b**) EAn, and porous morphology of (**c**) ECn and (**d**) EAn.

Pseudospherical expanded aluminum nodules (EAn) and quasi-spherical expanded clay nodules (ECn) were used as dispersed phases or fillers, as shown in Figure 1a,b. Both types of filler exhibit an internal porous morphology; in the case of EAn, porosity is closed-cell in contrast to ECn, which is opened-cell and commonly used in garden applications as a drainage product, as shown in Figure 1c,d. EAn is made of in-situ foaming precursor particles of 5 mm in height and 5 mm in diameter. These precursors are made of aluminum of commercial purity (99.5 wt%) and 10 wt% of white marble in powder (97.88 CaCO_3_, 1.3 MgO, 0.5 SiO_2_, and 0.1 others, in wt%), which were previously processed by P/M and hot extrusion [40,41]. ECn were provided by Fábrica Leca Portugal, S.A., Avelar, Portugal, in granulometry ranging from 10 to 20 mm in size. Chemical composition, according to company technical data is in wt%: 61.95 SiO_2_, 17.91 Al_2_O_3_, 8.18 Fe_2_O_3_, 3.90 K_2_O, 5.39 CaCO_3_, 1.38 MgO, 0.57 S, and other balances [21,42]. The main physical properties of both types of filler are collected in Table 1.

### 2.2. Methods

#### 2.2.1. Synthesis

There were two types of syntactic foams processed; the first type incorporates EAn, while the second type incorporates ECn. Based on the type of filler, the samples of syntactic foams are called Al-AEn and Al-Ecn. The ASFs were processed through MIT, and the infiltration of liquid metal was mechanically assisted by a ram in the direction of gravity force. The process was divided into 4 main steps: (1) A cylindrical steel mold of a pair of axisymmetric parts (85 and 37.5 mm in height and diameter) is cleaned with a metal brush to remove wastes from other processes. The internal and partial external surface of the part is coated with a solution of hexagonal bore nitride (HBN) and acetone. The HBN coating acts as a lubricant and protector to avoid the adherence of liquid aluminum to the mold’s internal surface and the incorporation of mold residues into the liquid metal. The fillers are cleaned in acetone for three minutes to remove organic residues. (2) The parts of the mold are assembled and held with the aid of a pair of stainless-steel clamps. The base of the mold is closed by a graphite disc of 10 mm in thickness and negligible tolerance. Fillers are poured into the mold and a disc of solid aluminum with a volume of 2/3 times the volume of fillers is located on them. (3) The whole system is located in the furnace set to reach a temperature of 100 °C over the melting temperature of the aluminum disc with a ramp of 1500 °C/H. A cylindrical sail of graphite, 37.5 mm in diameter, is placed on the solid matrix before turning on the furnace, which acts as thermal isolation, and the furnace is turned on. (4) After 40 min, the molten matrix is infiltrated into the open porosity between fillers aided by a mechanical ram that pushes the graphite sail. The velocity of infiltration is calibrated to operate at 2 mm/min. During the infiltration process, the gases into the open porosity of the bed of fillers are realized through the tolerance of graphite discs of the top and the base. The infiltration ends when a specific value of force is reached by the load cell. The mold remains inside the activated furnace during the entire infiltration process. The furnace is turned off, and the assembly is left to solidify inside for 15 min. Subsequently, the assembly is extracted and subjected to forced cooling.

Samples are sized following a ratio height-diameter of approximately one to meet the requirements of standard ISO-13314 [43]. Subsequently, they are subjected to a heat treatment of homogenization annealing, the parameters of which are shown in Table 2.

#### 2.2.2. Density and Porosity

Experimental density was determined based on its definition as the ratio of the weight of the samples and the cylindrical volume after being sized and heat-treated (Equation (1). The weight was registered with a Mettler Toledo PG403-S DeltaRange densimetric balance with an accuracy of 0.010 g. Theoretical density was computed according to the rule of mixtures (Equation (2)), and the volume fraction of fillers was estimated as a ratio between the volume of fillers and the volume of each sample (Equation (3)):

(1) $$\rho_{CMF}^{Exp}=\frac{m_{CMF}}{V_{CMF}},$$

(2) $$\rho_{CMF}^{Th}={\left[\rho \cdot f_{V}\right]}_{Matrix}+{\left[\rho \cdot f_{V}\right]}_{Filler}=\rho_{Matrix}\cdot \left(1-f_{V,Filler}\right)+{\left[\rho \cdot f_{V}\right]}_{Filler},$$

(3) $$f_{V,Filler}=\frac{V_{Filler}}{V_{CMF}}\cdot 100=\frac{\rho_{Filler}\cdot m_{Filler}}{V_{CMF}}\cdot 100,$$

Experimental porosity estimation requires the computation of the solid part volume fraction in the samples. This fraction is known as relative density, and it is equal to the ratio between samples and metal matrix densities. The subtraction of relative density to the volume of the samples represents the volume fraction of voids, which is known as total or experimental porosity (Equation (4)). The porosity inside the fillers incorporated in the samples can be estimated as the ratio between the volume fraction of the filler embedded and the porosity of the fillers, which is known as theoretical porosity (Equation (5)):

(4) $$P_{O,CMF}^{Exp}=1-R.D.=1-\frac{\rho_{CMF}^{Exp}}{\rho_{Matrix}},$$

(5) $$P_{O,CMF}^{Th}={\left[P_{o}^{Th}\cdot f_{V}\right]}_{Filler},$$

#### 2.2.3. Microstructural Analysis

For the microstructural characterization of the samples, a scanning electron microscopy (SEM) model FEI Quanta 400 FEG ESEM, from CEMUP, University of Porto, Porto, Portugal, was used. The semi-quantitative analysis was obtained by energy-dispersive X-ray spectroscopy (EDS). The samples were previously polished with SiC paper, followed by 6 and 1 µm diamond paste. The samples were then placed in an ultrasonic bath for 20 min and dried at room temperature.

#### 2.2.4. Mechanical Behavior

**Figure 2:**
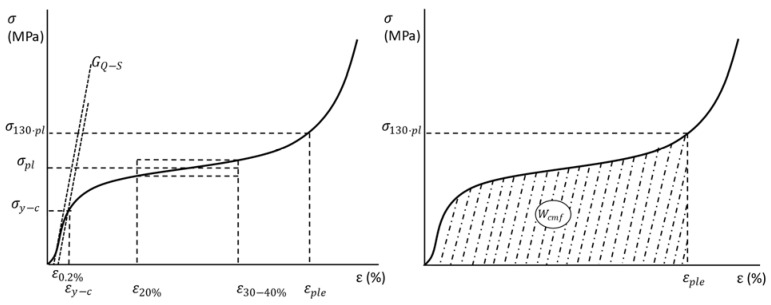
Graphical obtention of mechanical properties based on the stress–strain curve according to the standard used.

Both sets of samples were subjected to quasi-static (Q-S) compressive testing following the recommendations of ISO-13314 [43] for the study of cellular metal matrix materials. A universal testing machine was used to test the syntactic foams, Al-EAn and Al-ECn, and it was calibrated to operate at 2 mm/min (approx. 10^−3^ s^−1^). The elastic and plateau stages were registered by a load cell of 50 kN, while the densification was registered by a load cell of 300 kN. Based on the stress–strain curve, the following mechanical properties were computed (Figure 2): Quasi-elastic Slope (E*QS), compressive stress at 0.2% (σ_c_) as a substitute for yield stress; plateau stress (σ_pl_) as the average stress between 20 and 30–40% deformation or from the start to end deformation limits of the plateau stage; densification deformation (ε_ple_), which corresponds to the deformation at 130% of plateau stress, and finally, the absorbed energy (W), which is the area under the stress–strain curve between zero to densification strain.

## 3. Results

### 3.1. Manufacturing

According to ISO13314 [43], a minimum of three samples should be tested to meet the specifications. A total of five samples were produced in the case of the ECn-ASFs (Al-ECn), while three samples were synthesized in the case of the EAn-ASFs (Al-EAn) due to processing difficulties. In both sets of samples, the requirements of the standard were met.

**Figure 3:**
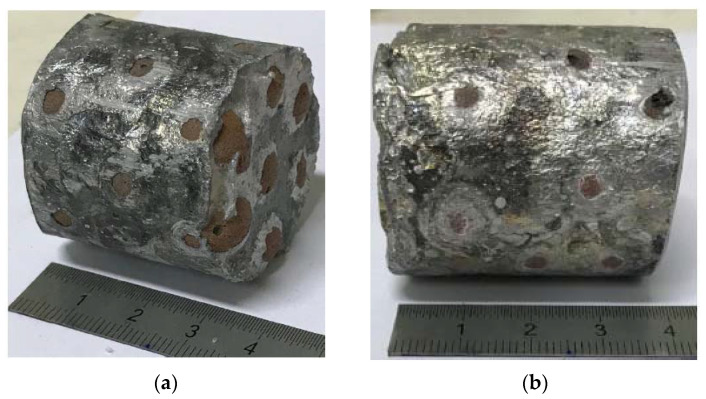
Sample of as-cast Al-ECn, (**a**) base and (**b**) profile.

It was observed that ECn exhibited brittle behavior under compressive loads, as was expected based on their ceramic composition. Previous studies [20,21,22] informed that beds of low thicknesses of these fillers can be successfully infiltrated through a maximum load of 1000 N without compromising the structural integrity of nodules. Thus, the infiltration stopping sensor was calibrated to stop the process when the load cell reaches this value. In addition, keeping in mind the brittle behavior of ECn, the graphite seal of the mold’s base was substituted by a soft seal of rock wool of 2 mm in thickness for damping load excesses. Consequently, the base of Al-ECn samples exhibited an irregular surface which has to be rectified (Figure 3a). The limitation in the infiltration load close to the maximum allowable load ensures an excellent infiltration, as can be observed in Figure 3b, in which voids between mold and fillers are almost coated by the matrix.

**Figure 4:**
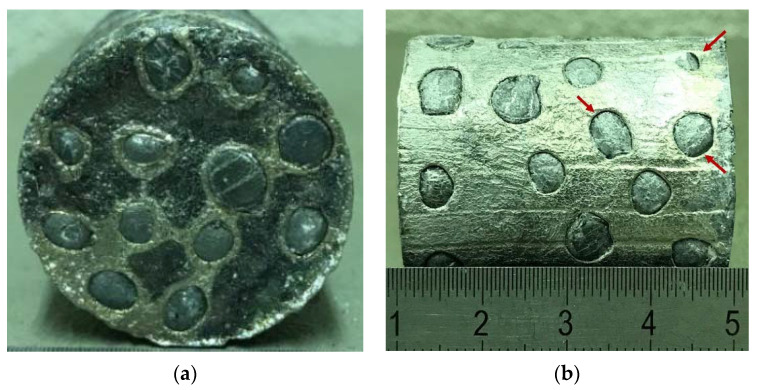
Sample of as-cast Al-EAn, (**a**) base and (**b**) profile.

The infiltration process of EAn is more restrictive than ECn due to the fact that the infiltration temperature is close to the melting temperature of the metal matrix of these fillers. Their behavior under compressive loads is ductile and similar to conventional metal foams. The temperature is so high that EAn could shrink under infiltration loads. However, the internal closed-cell porosity [40] and the development of an external coating of alumina during surface oxidation [44,45] limit this phenomenon. It was found through heuristic testing that loads higher than 5 N promote shrinkage of EAn. A hard graphite seal in the base of the mold was used to improve the homogeneity of the samples’ surface base, the results of which results can be observed in Figure 4a. The stopping displacement sensor that controls the infiltration was calibrated to stop at 5 N. This restrictive limit on the processing load promoted successful infiltration and more surface defects or voids, as can be observed in Figure 4b. Furthermore, there are some parallel striations as a consequence of irregularities in the HBN solid lubricant.

### 3.2. Density and Porosity

In Table 3, the computations relative to experimental and theoretical density can be observed. The volume fraction of incorporated fillers in both sets shows a slight difference of 4% in favor of Al-ECn, which means that the infiltration load for the expanded clay set of samples was more efficient. During the processing of Al-ECn, close to the maximum load of 1000 N the tolerances in the graphite seals from the top and base allow slight releasing of liquid metal excesses. Consequently, there is a lower volume fraction of the metal matrix in the samples, and the volume fraction of fillers increases proportionally. At the maximum infiltration load of 5 N (case of Al-EAn processing samples), excesses of liquid metal are not released through the tolerance of top and base graphite seals. Thus, these samples exhibit a slightly less average volume fraction of embedded fillers than Al-ECn. Nevertheless, the average volume fraction is higher than the minimum packaging for a theoretical random dispersion of fillers by 2% (52.3%) [6], which means that the maximum load and the volume of the metal matrix were appropriately selected. The volume fraction of infiltrated ECn tends to the maximum for a random close packaging of the same diameter spheres (64%), which supports the maximum selected [46]. It can also be observed that there are deviations between theoretical and empirical values of density in both sets of foams. Negative deviation in Al-EAn is due to deficiencies in the infiltration process, which is consistent with the low infiltration load selected. In contrast, positive deviation in Al-ECn is due to a relative excess of load during the last stage of the infiltration process. This deviation is negligible, demonstrating the efficiency of the limitation and density values according to previous studies of Al-ECn [20].

In Table 4, the results of the porosity analysis can be observed. Relative density (RD) was estimated as the ratio of experimental density and AA6063 density. Notwithstanding, Al-ECn exhibits higher incorporation of fillers than Al-EAn, and this set presents a higher average value RD. This apparent incoherence in the physical properties can be explained by analyzing the deviations in the expected porosity. Positive deviations from theoretical values imply the development of voids or deficiencies of infiltration. The residual porosity in Al-EAn is approximately 5%, which is close to the minimum fraction of voids developed by SCT [7]. The suitability of the maximum infiltration load of Al-ECn can be demonstrated by the limited residual porosity developed.

### 3.3. Microstructure

Previous studies [20,21,22] analyzed syntactic foams made of aluminum matrices, such as AlSi5Cu1Mg, AlSi7Mg, and AlSi9MgMn, embedding ECn, similar to those samples used in the present study as reference material. The microstructure of the interface matrix-filler in the mentioned studies showed a negligible chemical interaction between components promoting the so-called mechanical bonding.

**Figure 5:**
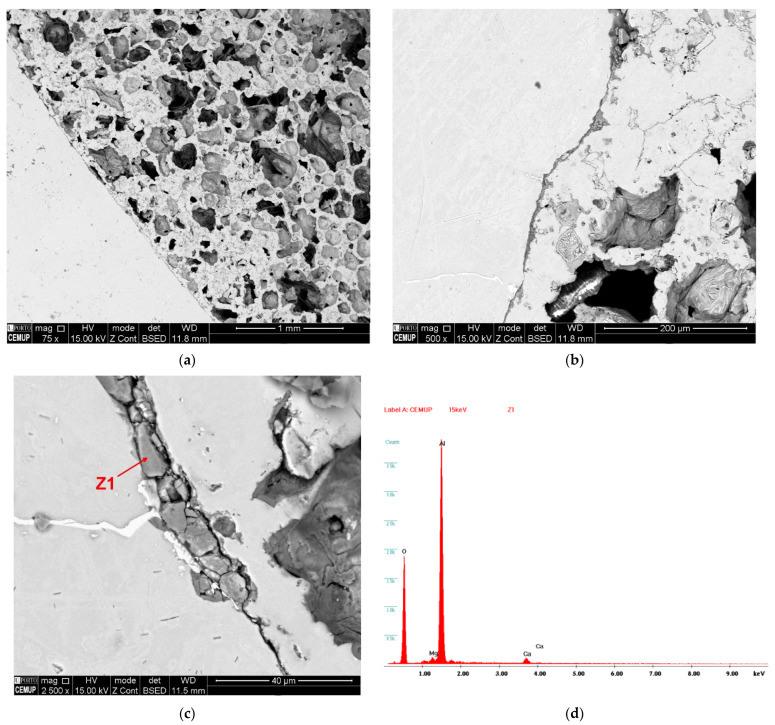
Scanning electron microscopy detail of Al-EAn post-homogenization: (**a**) porosity of EAn; (**b**) dark coating on EAn surface; (**c**) thickness of the oxide coating; (**d**) EDS analysis on the AlMg0.7Si–EAn interface.

In Figure 5a, it can be observed that EAn maintains the internal porosity after being embedded by the matrix despite the high temperature of the process. The internal morphology is constituted by irregularly rounded closed-cells of less than 1 mm, which is according to other studies of Al foams foamed with white marble [40]. A zoom on the interface matrix-filler (Figure 5b) reveals a thin dark coating that defines the frontier between components. The thickness of this dark layer is up to 10 µm under the processing conditions (Figure 5c). Previous research works [44,45] on the effects of heating cycles in an oxygen-rich atmosphere of aluminum surfaces demonstrated that temperatures higher than 300 °C promote the formation of thin coatings of aluminum oxide (alumina). Its thickness depends on the temperature and the exposition period. According to the EDS analysis (Figure 5d), the layer is mainly constituted of aluminum and oxygen, which confirms the presence of alumina. Low traces of calcium and magnesium around the alumina are due to the sublimation of white marble during the heating obtaining oxides. Thus, the interface AA6063-EAn is constituted by aluminum and alumina. In Figure 4b,c, a negligible interaction between EAn and the matrix can be observed, which means that the bonding between the matrix and fillers is strongly mechanical [47,48].

**Figure 6:**
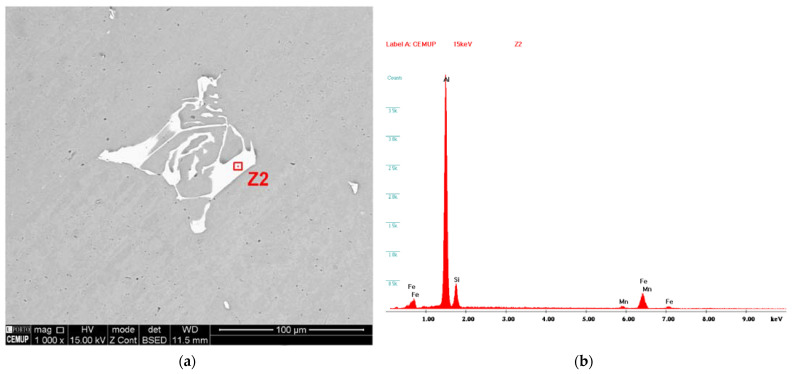
(**a**) Scanning electron microscopy detail of Al-EAn matrix area post-homogenization; (**b**) EDS analysis on the Chinese script.

Irregular intermetallics in the form of Chinese scripts can be observed in the matrix after being subjected to homogenization annealing (Figure 6a). During the heat treatment, the transformation of beta phases into alpha phases of AlFeSi takes place [49,50]. Precipitation of AlFeSi phases is confirmed through the EDS results shown in Figure 6b.

### 3.4. Mechanical Behavior

**Figure 7:**
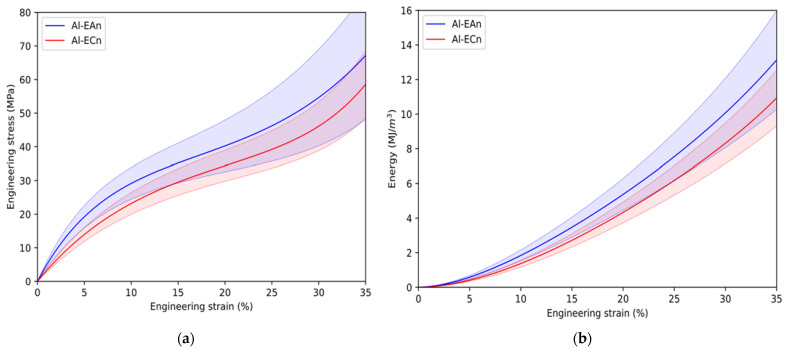
Average mechanical behavior of each series: (**a**) stress–strain curve and (**b**) energy absorption capacity.

Typical compressive stress–strain curves of MMSFs are classified into three main stages. In the first one, the MMSFs exhibit an elastic slope in which the matrix pillars and walls between fillers damp the loads without collapse. When matrix pillars collapse over the fillers, both matrix pillars and fillers increase the participation in absorbing mechanical energy. Subsequently, the porosity of the fillers starts to decrease proportionally to the increase in deformation and follows a quasi-constant slope. This second stage is known as a plateau, and it is characterized by a smooth and positive slope in which the porosity collapse dampens the mechanical energy until MMSFs reach an almost null porosity. This point is known as densification strain and represents the beginning of the third stage, the so-called densification. In this stage, MMSFs have lost their porosity, and their behavior shows a sudden increase in the resistance to compressive loads [20,46]. The slope of this stage is governed by the ductile or brittle behavior of the bulk material of the fillers. Based on the chemical composition of fillers, the plateau stage can exhibit three different evolutions: purely cellular, brittle, or ductile. In the case of purely cellular, the whole plateau stage exhibits a null slope until the densification strain is reached. In contrast, the plateau exhibits smooth changes of slope in each stage when fillers are ductile (metallic composition). Erratic and sudden changes of stress are common when the filler is brittle (ceramic composition) [1,2]. Figure 7a,b shows the average stress–strain and energy absorption curves and their standard deviation. The smoothness evolution of the curve of Al-ECn is in concordance with similar Al-ECn syntactic foam studied by other authors [22] for fillers 11 mm in size.

It can be observed that the average value of stress along the deformation of Al-EAn samples is slightly higher than the Al-ECn. This is due to the fact that the aluminum matrix of Ean dampens along with the elastic mechanical energy of the AA6063 matrix. This result is comparable to having more aluminum mass to absorb the mechanical energy during the elastic step than Al-ECn, and thus more stress is reached. Average values of elastic gradient and compressive stress were higher than Al-ECn (Table 5). From 5% to 10% of strain, both sets increase the damping of compressive loads by the fillers until almost the whole load is absorbed by fillers and the plateau stage begins. The porosity fraction of samples governs the extension of the plateau step, while other physical properties such as closed-cell, low sizes, and spherical shape of pores promote achieving more crushing stress under compression loads. The densification strain is reached around 30%, which is relatively close to the experimental porosity (Table 4).

**Figure 8:**
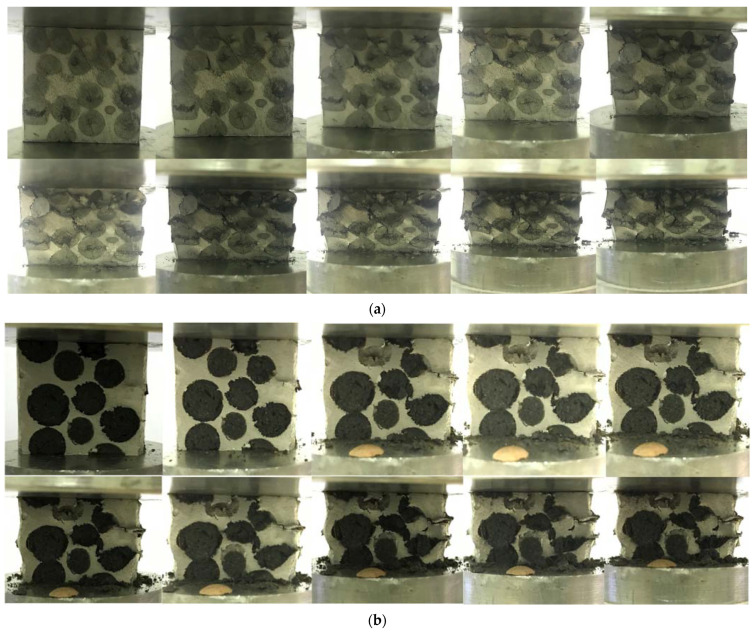
Deformation sequence from 0% strain in 5% steps from top left to right and down: (**a**) Al-EAn and (**b**) Al-ECn.

The effects of the deformation of Al-EAn are appreciable from 5% on the external surface of the sample (Figure 8a). The affected area corresponds to the surface of the bonding interface of the nodules and the matrix, where the bond is purely mechanical, and the absence of the matrix facilitates its displacement and deformation at the edge of the interface. Between 10 and 15%, collapse propagation is observed in the form of horizontal fractures from left to right, and it is initiated in nodules emerging from the cylindrical surface. The onset of the collapse is manifested in the head due to the slightly higher concentration of nodules. Above 15%, the EAn exhibited a repeated layer-by-layer deformation mode. This mode was also observed in previous studies by Linul et al. and Movahedi et al. [16,18] in SF embedding expanded perlite (EP) particles, which did not exhibit fragmentation after being tested. The ductile deformation mode is characterized by an increase in the middle height diameter concerning the original diameter; thus, the shape of the samples is similar to a deformed barrel, as shown in Figure 8a.

As expected according to Orbulov et al. [23], the failure mechanism of Al-ECn (Figure 8b) manifests a layer-by-layer mode governed by a uniform and continuous deformation. There were no distinguished shear bands at 45° concerning the compression direction as was observed by Movahedi et al., Majilinger et al., and Orbulov et al. with diverse particles [18,51,52]. The sample remained intact until the end of the testing.

**Figure 9:**
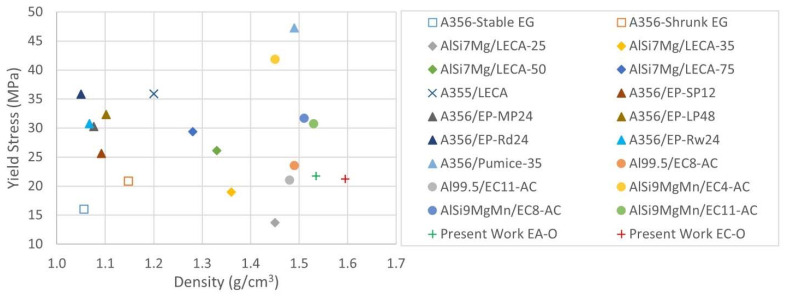
Comparison of average values of yield stress of ASF embedding porous fillers [11,12,13,14,15,20,21,22].

The average yield stress reached by both sets remains near the values obtained by other researchers in as-cast samples whose heat treatment state is quite similar to a homogeneous annealing treatment of the matrix, has a limited content of alloying elements, and for EC nodules of approximately 12 mm in size, as can be observed in Figure 9. Concerning EP and pumice syntactic foams, the present materials exhibit both inferior and slightly superior performance to stable and shrunk EG-Al syntactic foams.

**Figure 10:**
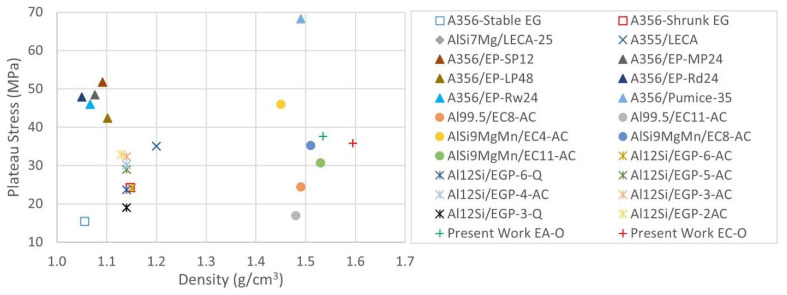
Comparison of average values of plateau stress of ASF-embedding porous fillers [11,12,13,14,15,20,21,22].

The deviation of Al-EAn on stress (the blue-filled area around the continuous curve) during plateau behavior exhibits higher amplitude than Al-ECn (Figure 7a). Since porosity governs this stage, the reason it causes higher deviation in the plateau stress is the residual porosity developed in the Al-EAn sample ranging from 2 to 8% in comparison to reference foams ranging from −5 to −3%. This hypothesis can be demonstrated by analyzing the amplitude of deviation values in the stages in which porosity is not an important parameter, such as the elastic or densification stages. In the elastic stage, it can be observed that low values of strain develop limited amplitudes of deviation, and these do not overlap themself in contrast to the plateau stage in which porosity governs mainly the behavior of the samples. The average values of plateau stress show a limited difference of around 2 MPa in favor of Al-AEn (Table 5). In Figure 10, it can be observed that the plateau stress value of Al-ECn is close to values found for similar Al99.5-LECA11 SF [22]. The gradient of the plateau is almost the same for both sets, according to Figure 7a. However, higher densification strain is observed in average values in favor of Al-ECn with a difference of 3%, and differences of around 2 MPa are observed in favor of Al-EAn in the case of densification stress average values.

**Figure 11:**
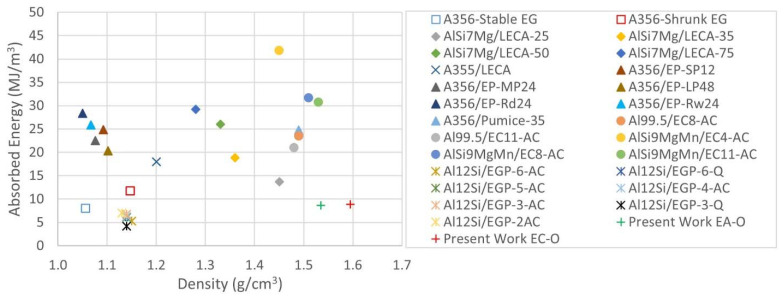
Comparison of average values of energy absorption capacity of ASF-embedding porous fillers [11,12,13,14,15,20,21,22].

The evolution of absorbed energy of both sets of ASF can be observed in Figure 7b; Al-EAn exhibits a difference of almost 5% of absorption energy compared to Al-ECn at a strain of 35%. This is the strain that was reached for the maximum load of the load cell and the strain at which the testing machine stopped the displacement. Interestingly, the average values of absorbed energy are close, exhibiting 8.615 (Al-EAn) and 8.865 MJ/m^3^ (Al-ECn), as shown in Table 5. In comparison to other studies shown in Figure 11, Al-AEn and Al-ECn show energy values in the range of EG ASF and slightly less than AlSi7Mg-LECA syntactic foams [14,20].

The above-mentioned mechanical and physical properties demonstrate that Al-EAn syntactic foams are potential substitutes in those applications in which Al-ECn can be applied. Furthermore, these are particularly interesting when recyclability is a limiting parameter (because of the high recyclability of aluminum), and it is required to reduce the exploitation of natural resources and economical costs using mining wastes as raw materials.

## 4. Conclusions

Aluminum is one of the metals with the highest recyclability, low density, and high richness in the earth’s crust and can be used in a wide variety of products in sectors such as aerospace, transportation, construction, defense, etc. Due to the need to manufacture materials with a high degree of recyclability and that allow energy savings in transportation, it is essential to develop new materials that meet these needs. From the analysis of physical properties, mechanical characteristics, and microscopy analysis, it is concluded that:

- It is feasible to synthesize Al–Al syntactic foams using nodules with porous closed-cells and fine internal morphology by melt infiltration routes. These nodules are made of aluminum of commercial purity and were foamed with wastes of marble from ornamental rock quarries as a low-cost substitute for conventional titanium hydride.
- Al-EAn have an average relative density of 56% and an average total porosity of 43%. The average residual porosity is approximately 5%. No significant chemical interaction is observed at the nodule–matrix interface; thus, the bonding in the interface is purely mechanical.
- The Al-EAn samples exhibit similar mechanical properties to the Al-ECn samples, although slightly higher due to EA nodules exhibiting a higher crush load than EC nodules. The low volume fraction of EAn compensates for the higher crush load concerning ECn. The 5% residual porosity of the Al-EAn samples manifests itself in a higher standard deviation of the porosity-governed properties: plateau stress and densification strain. Samples with Al-Ean have approximately the same energy absorption capacity as samples with Al-ECn.
- The stress–strain curves demonstrate the ductile nature of the matrix. The samples with nodules of EC show slight stress spikes along with the transition between the elastic stage and the plateau, demonstrating the brittle nature of the ECn. The Ean series does not experience any spikes, evidencing the ductile nature of the dispersed phase, and the effect of the alumina film coating of the nodules on the mechanical behavior is negligible.
- The deformation mechanism that governs both materials is ductile because both exhibit a uniform and continuous deformation layer-by-layer and a slight barreling phenomenon.

In the homogenization annealed state, Al-EAn can be used in applications where a low-density material with high recycling potential and similar values of yield strength (20–25 MPa) and plateau stress (30–40 MPa) to Al-ECn are required.

## Figures and Tables

**Table 1:** Physical properties of aluminum and porous clay fillers.

Space Holder	Density kg/m^3^	Height mm	Diameter mm	Crush Load N	Porosity %
Aluminum (EAn)	820	7–8	8.5	365	70
Clay (ECn)	800	NA	11.5–12.5	308	80

**Table 2:** Parameters of the homogenization annealing of AA6063 [39].

Alloy and Heat Treatment	Temperature (°C)	Time (min)	Cooling Rate (°C/Hour)	Temperature (°C)	Rate (°C/Hour)	Temperature (°C)
6063-O	415	180	−28	260	Natural	Room

**Table 3:** Average values of physical properties of rectified and heat-treated ASFs.

Set of Samples	Weight (g)	H/D Ratio (-)	Experimental Density (g/cm^3^)	Volume Fraction of Fillers (%)	Theoretical Density (g/cm^3^)
Al-EAn	60.65 +/− 3.591	0.954 +/− 0.029	1.535 +/− 0.086	54.464 +/− 0.687	1.680 +/− 0.013
Al-ECn	52.838 +/− 2.736	0.8 +/− 0.027	1.595 +/− 0.039	58.294 +/− 2.629	1.586 +/− 0.051

**Table 4:** Average values of porosity parameters of rectified and heat-treated ASFs.

Set of Samples	Relative Density (%)	Experimental Porosity (%)	Theoretical Porosity (%)	Residual Porosity (%)
Al-EAn	56.836 +/− 3.167	43.164 +/− 3.167	38.021 +/− 0.479	5.142 +/− 3.086
Al-ECn	59.074 +/− 1.431	40.926 +/− 1.431	44.758 +/− 2.018	−3.832 +/− 1.084

**Table 5:** Average and deviation of mechanical properties estimated based on the guidelines of the ISO-13314 standard [31].

Set of Samples	Elastic Gradient (MPa)	Yield Stress (MPa)	Yield Strain (%)	Plateau Stress (MPa)	Densification Stress (MPa)	Densification Strain (%)	Absorption of Energy (MJ/m^3^)
Al-ECn	343.309 +/−51.012	21.247 +/−5.083	6.883 +/−0.918	35.84 +/−6.599	46.592 +/−8.579	30.136 +/−2.254	8.865 +/−2.067
Al-EAn	521.030 +/−60.802	21.742 +/−5.181	4.282 +/−0.371	37.647 +/−2.957	48.942 +/−3.844	27.206 +/−5.359	8.615 +/−0.676

## Data Availability

Not applicable.
